# COINS Data Exchange: An open platform for compiling, curating, and disseminating neuroimaging data

**DOI:** 10.1016/j.neuroimage.2015.05.049

**Published:** 2015-05-24

**Authors:** Drew Landis, William Courtney, Christopher Dieringer, Ross Kelly, Margaret King, Brittny Miller, Runtang Wang, Dylan Wood, Jessica A. Turner, Vince D. Calhoun

**Affiliations:** a The Mind Research Network, 1101 Yale Blvd NE, Albuquerque, NM, USA; b Department of Psychology, Georgia State University, Atlanta, GA, USA; c Department of Electrical and Computer Engineering, University of New Mexico, Albuquerque, NM, USA

**Keywords:** Neuroinformatics, Data sharing, Data Exchange, DX, COINS

## Abstract

Neuroimaging data collection is inherently expensive. Maximizing the return on investment in neuroimaging studies requires that neuroimaging data be re-used whenever possible. In an effort to further scientific knowledge, the COINS Data Exchange (DX) (http://coins.mrn.org/dx) aims to make data sharing seamless and commonplace. DX takes a three-pronged approach towards improving the overall state of data sharing within the neuroscience community. The first prong is compiling data into one location that has been collected from all over the world in many different formats. The second prong is curating the data so that it can be stored in one consistent format and so that data QA/QC measures can be assured. The third prong is disseminating the data so that it is easy to consume and straightforward to interpret. This paper explains the concepts behind each prong and describes some challenges and successes that the Data Exchange has experienced.

## Introduction

Neuroscience increasingly requires larger and larger datasets in order for scientists to draw statistically significant conclusions. Unfortunately, collecting this data is both monetarily expensive and time consuming. In addition to the cost and time barriers, once data has been collected, studies are often reluctant to share this data for fear of losing their competitive edge by sharing the data before they are able to fully analyze and publish on it themselves. This waiting period creates missed opportunities during which time others in the field may have been able to discover new breakthroughs by analyzing the data. Also, others could be duplicating effort by collecting similar data that they may not have needed to collect if they had access to the already collected data (Milham, 2012). These examples represent both technical and cultural barriers to data sharing. Some of these barriers (cost and time) can be overcome with technical solutions. Other barriers (reluctance to quickly share data) may require a cultural shift in the neuroscience community in addition to a technical solution. The Data Exchange (DX) was created in an effort to help overcome technical difficulties associated with sharing neuroscience data.

Data Exchange is an open source data sharing initiative that has been created by developers at The Mind Research Network (MRN) in conjunction with the study management software Collaborative Informatics Neuroimaging Suite (COINS) (Scott et al., 2011; King et al., 2014). COINS currently hosts more than 550 studies from 18 sites spread throughout the United States. COINS contains more than 400,000 cognitive assessments and 350,000 functional MRI images, from 30,000 research participants. COINS is made up of many different modules, that when combined, form a suite of software used for end-to-end study management specifically targeted at neuroscience data. Some of these modules are:

- *MICIS*, which manages participant details (PII, etc.), study details (IRB details, expiration dates, number of allowed participants, etc.), and scan information (scan time, scanner type, location of DICOM files in the archive, etc.).
- *Assessment Manager*, which is used for building assessment templates and collecting clinical assessment data.
- *Query Builder*, which is used for querying clinical assessment data and generating exportable reports in an anonymized fashion that protects individual participant PII.
- *Participant Portal,* which is a web front end that allows research staff to create a template-able schedule of assessments for specific participants such that those participants are able to complete assessments from their own home computer so that research staff do not have to personally administer every assessment on site. This *schedule of assessments* can be a one-time event or a queue of separate events. These assessment events are given a specific start and end time. Participant Portal only shows participants the assessments that are available during the time when the participant logs in.
- *Data Exchange,* which is used for managing all events related to data sharing. DX is currently sharing 55,000 cognitive assessments and 16,000 functional MRI images collected from 4100 research participants.

All of the COINS tools can be accessed via the internet using modern web browsers (Wood et al., 2014).

Data Exchange was designed to share neuroscience data. The three main parts that make DX an effective data sharing platform are data compilation, data curation, and data dissemination.

## Framework

### Data compilation

The first goal of DX is to provide a system that compiles the data that researchers have collected. Since DX is a module contained within the COINS suite of software tools, DX is able to take advantage of the data management infrastructure that has already been built into COINS. Any data that was collected using COINS, and thus already stored within COINS, can easily be shared via DX with just the click of a button. On the other hand, if data is not already stored in COINS, there are other avenues for uploading that data ad-hoc and making it available to share.

This ad-hoc data upload process is somewhat more involved, but not difficult. The data upload tool first requires that a researcher fill in basic information about their study (e.g. site name, location, study name, scanner details, etc.). Once those details are filled in, they can then begin uploading the data, which is validated and archived using automated processes developed by the COINS team. Once the process is complete, data is shareable within DX as if it had been natively collected using the COINS framework.

There are multiple datasets currently being shared via DX that were collected at various sites around the world using distinct collection strategies and approaches and then uploaded to DX using the ad-hoc data upload process.

Data Exchange was built with the idea of being able to host many different types of data. Currently DX serves both pre-processed and raw DICOM data, as well as behavioral data. Here are a few examples of formats that DX is currently serving: .bval, .bvec, .csv, .dat, .dcm, .edat, .gz, .hdr, .img, .info, .mat, .nhdr, .nii, .nrrd, .raw, .txt, and .zip. Theoretically, there is no limit to what type of data DX can serve.

Data providers are free to choose what format they would like to share their data in and if they want to do any form of data manipulation prior to sharing. Some data providers have chosen to deface DICOM images prior to sharing them. Others have not defaced their DICOM images. Prior to zipping data into containers for download, automated processes anonymize sensitive DICOM headers in order to remove identifying information regarding the research participant.

The COINS architecture provides a dynamic interface between the data storage system and the data retrieval system. This gives DX the flexibility to easily include and share new data types. In addition to sharing MRI scan data and cognitive assessment data, data providers will also be able to share MEG, EEG, and genetic data.

### Data curation

Once DX has compiled the data and organized it into a meaningful structure, the data must then be curated in order to guarantee that it is research quality. DX contains data that spans the entire lifecycle of a study — ranging from studies that are actively collecting to studies that are no longer collecting. It is understood that as time progresses, data that was initially thought to be research quality, may become disqualified. For example, there may be a research participant that becomes excluded from a study or there may be a scan sequence that was run incorrectly and the mistake is only discovered after the data was already shared. DX is able to handle these situations where data was initially included and then after fact it becomes known that the data is not research quality with fine-grained controls that allow for data to be excluded ad-hoc. In addition, study administrators are also able to use DX for communicating with users that have downloaded the problem data. This allows them to explicitly tell users that a piece of data they have downloaded should no longer be considered research quality.

**Fig. 1:**
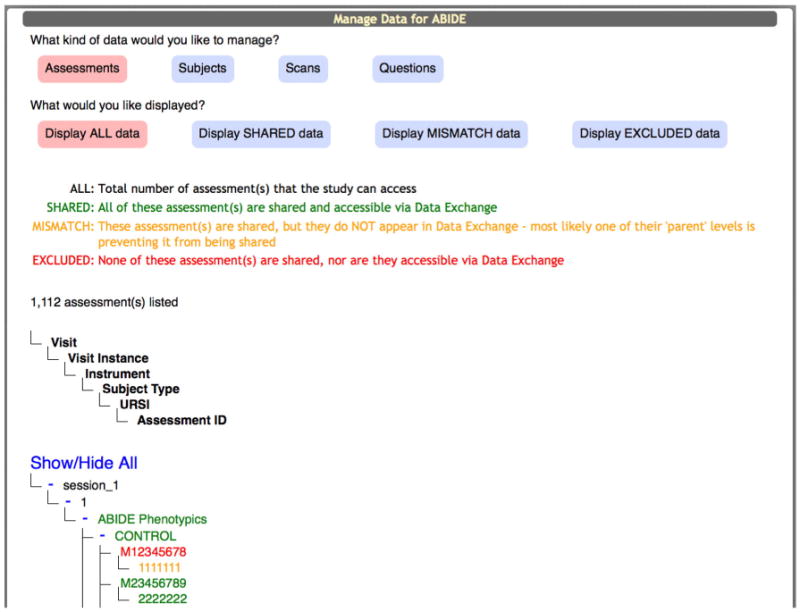
Study management page.

Data providers and COINS developers collaboratively maintain data quality. Data providers can curate their own data using built-in management tools. The COINS team is also available to assist them when necessary. Fig. 1 shows the study management page that gives study administrators fine-grained control over exactly which data they want to share and which data they do not want to share.

Data providers are free to include more data (or exclude data) even after the study has been shared in DX and data requesters have already started to request and download data. If data is excluded, DX is able to recognize that the data should no longer be shared and will not zip that specific data with any future requests. In addition to excluding data for all requests moving forward, DX is able to search all previously requested data and recognize which previously built data capsules contain data that has now been excluded. Data downloaders can then look at their previous requests and see if any of their requested data has been excluded.

### Data dissemination

One of the main barriers to science in the 21st century is not the lack of data, but rather being able to find data that will actually be meaningful to the project at hand. Therefore, the third goal of DX is to disseminate the data in a way that is easily searchable and easily understandable.

DX provides an easy to use graphical user interface (GUI) for data requesters that allow them to pinpoint the exact data that they are looking for. Large-scale neuroimaging data download has costs directly associated with it. In addition to the bandwidth and storage costs, if too much data has been downloaded, then researchers need to manually sift through enormous datasets and manually determine exactly what data is useful and what is not. DX encourages data downloaders to use a targeted approach by making it easy to see what data is available and what data is applicable. In a sense, this encourages a precise query, followed by a targeted download, as opposed to the shotgun method of blindly downloading as much data as is available.

The process for downloading data from DX is simple. A data requester first creates a targeted query using the GUI. Searching and requesting data is open and available to everyone. A COINS account is required, but anyone with an email address can sign up for free at http://coins.mrn.org/dx. Once a user has created an account, both imaging and phenotypic data are accessed in the same way.

**Fig. 2:**
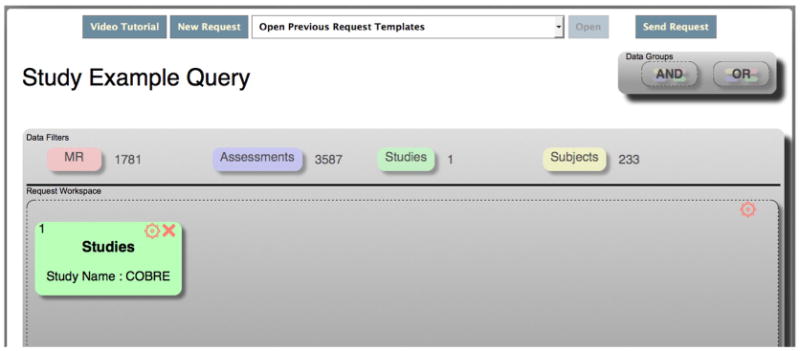
DX query for all data from the MRN COBRE study.

Fig. 2 shows an example of one of the simplest queries available. For example, if a data requester knew that they were looking for all the data from the MRN COBRE study (both cognitive assessments and functional MRI images), this is what the query would look like. This screen shot shows that there are 1781 functional MRI images and 3587 assessments available from 233 unique research participants.

**Fig. 3:**
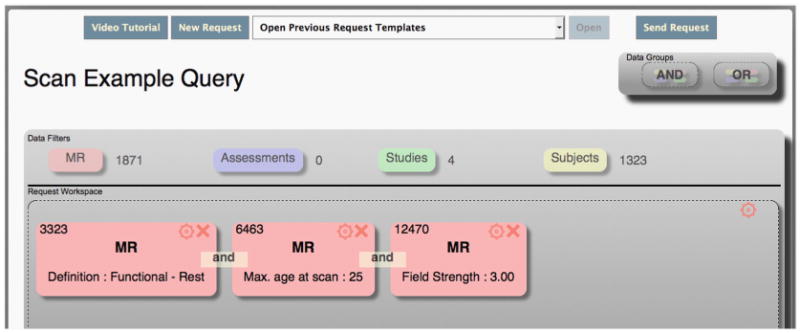
DX targeted query for specific scan data.

Alternatively, if a researcher is not looking for data that comes from a specific study, they could instead create a more targeted query based on the *type* of data included. Fig. 3 shows a specific query that only returns functional resting state scans from 3.0-Tesla scanners for participants that were no more than 25 years old at scan time. This query shows that DX contains 1871 functional MRI images from 1323 unique research participants collected from four separate studies.

Once a data requester has found the data that they would like to download, they then click the “Send Request” button and are required to agree to any pertinent Data Usage Agreements (DUA). DX allows each study to define its own DUA. In order to download data from a study, requesters must first agree to the most recent version of the DUA for those data. DX automatically tracks DUAs and prevents users from requesting data until *after* they have agreed to pertinent DUAs. If a requester has agreed to a DUA in the past, then they will not be asked to agree to it again. However, if a requester agrees to a DUA and then the study updates the DUA, the next time that the data requester requests data from that study, they will need to agree the newest version of that DUA. Each study that shares data within DX assigns a person from its group to function as the study administrator. Study administrators are able to see all data requests that include data from their study, they can communicate with any data requester that requests data from their study (via tools built into DX), and they can approve or deny requests. By allowing study administrators the ability to approve and deny requests, data providers have the ability to decide on a case-by-case basis who can access their data and for what purpose they can use it. If a study does not want to individually approve data requests, they can also make their data “pre-approved”. By making data pre-approved, any data requester can query the data, agree to DUAs, and then immediately download the data without needing to first gain manual approval from the study administrator.

After study administrators approve a request, DX then begins to process that request by anonymizing the data, zipping that data into small chunks (∼10 GB in size), then serving that data to the data requester. This packaging system enables custom downloads and distinguishes DX from other tarball-only approaches that may require the data requester to download the entire dataset instead of just the specific data that they are looking for.

Different studies that are sharing data within DX have different levels of access that they are willing to provide with respect to their data. For example, the Autism Brain Imaging Data Exchange (ABIDE) dataset has pre-approved their entire dataset (Nooner et al., 2012). When a user requests the data and agrees to the DUA, the data is immediately anonymized, packaged, and made ready for download—all in an automated process. On the other hand, some studies (and some IRBs) prefer to have more control over who is able to access and utilize their data. This increased level of control is gained by requiring that data requests be explicitly approved by study staff before any data can be downloaded. This gives data providers the opportunity to gather additional information and legal documents from the requester. For instance, some studies also require that the data requester provide their institution name and a brief description of how they plan to use the data.

In addition to the above access controls, DX also has a built-in messaging tool that allows the data requester and the study administrator to communicate with each other directly if clarification on anything additional is required. One, or all, of these access controls can be used in order to more closely manage who is able to access the data. Data providers are free to choose how closely they would like to monitor and manage their data sharing.

DX also includes a study information page that allows the data provider a means for including additional high-level information about the study along with useful study documents.

All participants that are downloaded through DX have a uniquely identifying number associated with them that is the same across all downloads. This allows researchers from different institutions to be able to reference specific scans, or specific cognitive assessments from a specific participant, and then researchers from another institution can find and download that same data.

## Results/Conclusion

Currently, DX is implicitly linked to the COINS architecture and hosted solely by MRN. At the moment, in order to share imaging data, that data must first be uploaded to MRN's local servers. However, this is different from the way that COINS' data collection functions in general. COINS users have multiple options for collecting data. These options include both transferring data to MRN's local servers and/or locally storing and managing data onsite themselves. MRN is currently in the process of creating a standalone DX package that would allow researchers to host their own data sharing platform. This would mimic the flexibility provided through COINS by allowing data providers to keep all their data onsite and to monitor the data themselves more closely.

DX has been able to successfully and effectively improve the state of data sharing in the world today by creating a means for uniting data providers and data consumers from all over the world. DX gives the data provider a simple set of tools and interfaces to have fine-grained control over exactly which data they are sharing. Data consumers, likewise have a simple set of tools that they can use for querying the data, requesting the data, agreeing to data usage agreements, and downloading the data. End-to-end, the whole process is contained within DX in one easy to use package.

To date, there are more than 600 DX users that have queried the database and searched for data. DX has packaged more than 31 TB of data coming from almost 1200 unique data requests.

**Fig. 4:**
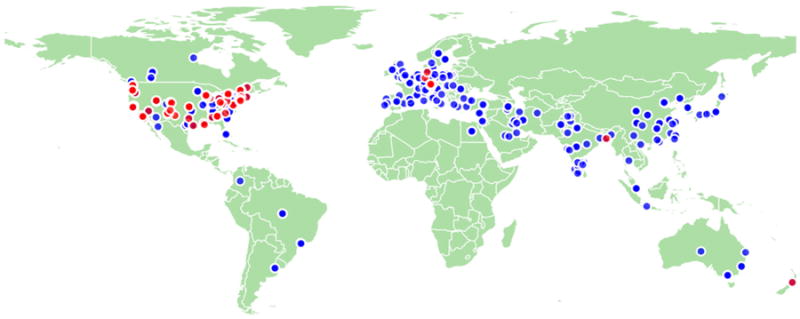
Data map showing locations where data requesters (blue) and data providers (red) have logged in.

Fig. 4 shows a map of all the locations where data requestors (blue) and data providers (red) have logged in from around world.

While the price of data storage and data serving has come down drastically in the last decade, there are still costs associated with being a data provider. In order to guarantee that DX can continue to be developed and maintained as a future resource, its costs have been spread out over multiple funding sources. We are thankful for support over the years from external funding agencies (NIH, DOE, NSF), customers who are using COINS to capture and manage data, as well as to MRN for generous institutional support.
